# The effects of clinical decision support system for prescribing medication on patient outcomes and physician practice performance: a systematic review and meta-analysis

**DOI:** 10.1186/s12911-020-01376-8

**Published:** 2021-03-10

**Authors:** Sharare Taheri Moghadam, Farahnaz Sadoughi, Farnia Velayati, Seyed Jafar Ehsanzadeh, Shayan Poursharif

**Affiliations:** 1 Department of Health Information Management, School of Health Management and Information Sciences, Iran University of Medical Sciences, Tehran, Iran; 2 Health Management and Economics Research Center, School of Health Management and Information Sciences, Iran University of Medical Sciences, Rashid Yasemi Street, Vali-e Asr Avenue, Tehran, 1996713883 Iran; 3 School of Health Management and Information Sciences, Iran University of Medical Sciences, Tehran, Iran; 4 Faculty of Medicine, University of Alberta, Alberta, Canada

**Keywords:** Computerized clinical decision support systems, Medication prescription, Systematic review

## Abstract

**Background:**

Clinical Decision Support Systems (CDSSs) for Prescribing are one of the innovations designed to improve physician practice performance and patient outcomes by reducing prescription errors. This study was therefore conducted to examine the effects of various CDSSs on physician practice performance and patient outcomes.

**Methods:**

This systematic review was carried out by searching PubMed, Embase, Web of Science, Scopus, and Cochrane Library from 2005 to 2019. The studies were independently reviewed by two researchers. Any discrepancies in the eligibility of the studies between the two researchers were then resolved by consulting the third researcher. In the next step, we performed a meta-analysis based on medication subgroups, CDSS-type subgroups, and outcome categories. Also, we provided the narrative style of the findings. In the meantime, we used a random-effects model to estimate the effects of CDSS on patient outcomes and physician practice performance with a 95% confidence interval. Q statistics and I^2^ were then used to calculate heterogeneity.

**Results:**

On the basis of the inclusion criteria, 45 studies were qualified for analysis in this study. CDSS for prescription drugs/COPE has been used for various diseases such as cardiovascular diseases, hypertension, diabetes, gastrointestinal and respiratory diseases, AIDS, appendicitis, kidney disease, malaria, high blood potassium, and mental diseases. In the meantime, other cases such as concurrent prescribing of multiple medications for patients and their effects on the above-mentioned results have been analyzed. The study shows that in some cases the use of CDSS has beneficial effects on patient outcomes and physician practice performance (std diff in means = 0.084, 95% CI 0.067 to 0.102). It was also statistically significant for outcome categories such as those demonstrating better results for physician practice performance and patient outcomes or both. However, there was no significant difference between some other cases and traditional approaches. We assume that this may be due to the disease type, the quantity, and the type of CDSS criteria that affected the comparison. Overall, the results of this study show positive effects on performance for all forms of CDSSs.

**Conclusions:**

Our results indicate that the positive effects of the CDSS can be due to factors such as user-friendliness, compliance with clinical guidelines, patient and physician cooperation, integration of electronic health records, CDSS, and pharmaceutical systems, consideration of the views of physicians in assessing the importance of CDSS alerts, and the real-time alerts in the prescription.

## Background

The health care industry is influenced by factors that increase costs and reduce the quality of health services [1]. One such consideration is the prescribing errors and drug interactions that are common among medical errors; hence, there is no need to note that avoiding such errors is of the utmost importance in preventing the side effects of drugs and other related consequences [2]. One of the most important medical errors that can lead to morbidity, mortality, and prolonged hospital stay is an inappropriate prescription medication [3]. Owing to a lack of clear documentation of medical history as well as data recording and reporting systems, the primary explanation for most prescription errors is insufficient knowledge about patients or their drugs [4]. The Clinical Decision Support System (CDSS) technology is also commonly used in the field to decrease prescription errors through reminders and alerts; meanwhile, it improves physician performance and patient outcomes [5]. On the basis of patient circumstances, CDSS is used to coordinate complex activities from initiation to monitoring and completion of medical care as well as providing guidance to physicians [6].

Various types of CDSS systems based on clinical guidelines, alerts, reminders, instructions, and recommendations are included in this study. For instance, the alert-based type of CDSS uses reminders and drug interaction alerts [7]. CDSS benefits involve reducing prescribing errors by using alerts and immediate reminders, automated dosing error checks, and drug interactions. E-prescribing systems with support for clinical decision-making have the potential to decrease errors and improve clinical practice [8]. The assessment of the effects of all computerized health care interventions is important in managing the health care process and patient outcomes [9]. Over the past years, a number of systematic studies have been conducted with the goal of analyzing the effect of CDSSs on prescription errors or CPOEs on patient safety, the care process, or the performance of physicians. In 2003, a systematic review of the two major databases revealed a reduction in drug errors due to the use of CDSS; however, the specifics of the findings have not been disclosed [10]. Another systematic review was also conducted in 2008 with an emphasis on the effects of CPOEs on medication errors. The results of this study showed a decrease in risk failure errors in 23 out of 25 included studies. While demonstrating the effectiveness of CPOEs, this research did not explain the outcome of the patients [11]. In the same way, another systematic review examined the effect of CDSS on prescribing errors in 2010. Since this analysis omitted the Randomized Controlled Trial (RCT) tests, the findings indicated a small change in the patient outcome. However, there has been a significant improvement in the care outcome process [3]. In another study in 2015, a review of the systematic reviews of the CDSS on patient safety was conducted. The results of this study showed improvement in outcomes. However, the authors argued that they need to include more studies with greater data pools in order to be able to further validate the CDSSs effect on outcomes [12]. In this systematic review, the most recent sample was collected in 2014 on a limited medication laboratory domain for some particular diseases [13]. In addition, another systematic study was carried out in 2017 to evaluate the effects of various forms of alerts on patient safety and medical outcomes. Surprisingly, the findings of the study showed no significant difference between various types of alerts except for some interrupting alerts that did not contribute to any improvement in outcomes [14]. CPOE was used for pediatrics in another systematic study whose purpose was to determine the errors. Results of this study demonstrated the usefulness of the system [15].

Considering the literature we have reviewed so far, the results of most studies have indicated the efficacy of CDSS compared to conventional clinical practices. The literature on CDSS has also shown progress in the physicians’ efficiency; however, the effect of these programs on patient outcomes is still uncertain [3, 16–18]. Due to the fact that CDSSs have been verified as useful tools to reduce prescribing errors, we decided to consider all types of CDSSs for all diseases and patients since 2005. Given the importance of CDSSs, in the present study, we examine the effects of CDSSs on physician prescribing performance and patient outcomes.

## Methods

We used a systematic review and meta-analysis in this study. The method section is divided into a variety of subsections, including search strategy, inclusion/exclusion criteria, screening and data extraction, quality assessment, data synthesis, and statistical analysis. Each subsection is described in more detail, as follows.

### Search strategy

The initial search was performed in PubMed to identify the keywords. We used Medical Subject Headings (MeSH) in PubMed, Emtree in Embase, and other words/phrases used in related papers as the basis for a search strategy. The major search was then conducted in PubMed, Embase, Web of Science, Scopus, and Cochrane Library. We performed the search in 2018 and used an approach tailored for each database without any language restrictions. Alerts were used to access published papers after the search date, and all database alerts were checked until July 2019. Reference tracking and citation search were also used to improve the retrieval of eligible studies. An example of the complete search strategy is given below:

(("clinical decision support system*" OR "clinical Decision Support*" OR "computerized decision support tool*" OR "Information System*" OR "computerized physician order entry*" OR "hospital information system*" OR "computerized medical record system*" OR "point-of-care system*" OR "medical order entry system*" OR "computer-assisted decision making" OR "computerized medical record system*" OR "reminder system*" OR "computer-assisted diagnosis" OR "clinical informatics*")) AND ("medical mistake*" OR "medical error*" OR "therapeutic error*" OR "diagnostic error*" OR "drug interaction*" OR "drug dose–response relationship" OR "drug administration schedule" OR "drug monitoring").

Registration number on PROSPERO is CRD42018079936 [19].

### Inclusion/exclusion criteria

We used the PICO criterion to conduct the search strategy: Participants (P) were individual practitioners or graduate trainees (e.g. medical residents); intervention (I) was any form of CDSS/CPOE system applied to the prescribing process; comparator (C) were those papers that used other systems or did not use any system; outcome (O) was any patient outcomes and physician performance outcomes. In this study, we included randomized CDSS clinical trial papers such as alert-based, recommendation-based, instruction-based, and reminder-based systems to assess their effects on patients and providers. In selecting a paper for this study, we first prepared a list of questions whose answers form the key criterion for inclusion as follows:

- Does the research concentrate on assessing the prescribing CDSS/CPOE based on any category of patient outcomes and physician performance outcomes?
- Is the study a randomized clinical trial in which the patient care was compared with and without prescribing CDSS/CPOE?
- Have experts such as physicians, specialists, and residents used the CDSS for prescribing CPOE in these studies?
- Does the decision support system/CPOE evaluate patient-specific information in the form of management or likelihood choices or recommendations for physicians?
- Has the practice been identified as a measure of the improved care process or the outcome of patients with any improvement in the study?

We excluded non-experimental studies as well as the studies in which the system was used exclusively by students who were not experts, or "no" was given as the answer to these five main questions.

### Screening and data extraction

**Fig. 1:**
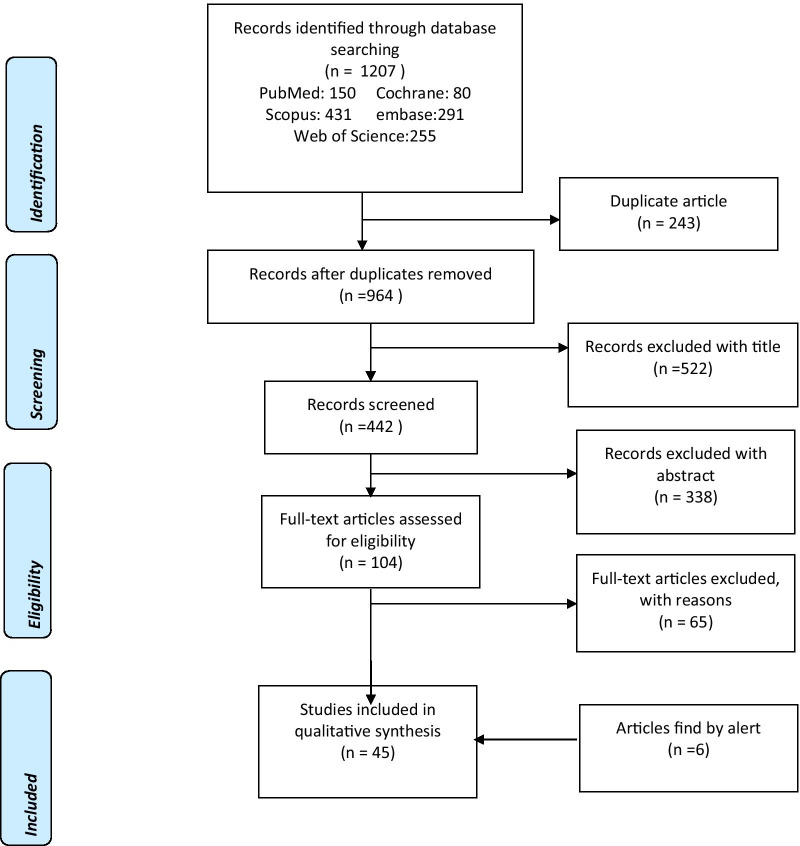
The PRISMA flow diagram of selected studies. The number of records for each database is specified. The PRISMA theory approach is also displayed in the blue rectangles

The papers were screened in three separate steps based on title, abstract, and full text. In the meantime, we used the Preferred Reporting Items for Systematic Reviews and Meta-Analyses (PRISMA) checklist as a reporting guide-line in our study. The results of the search are shown in Fig. 1. PRISMA checklist is a well-established standardized checklist for systematic review studies [20]. The evaluation was carried out by two authors of this study (S.T) and (F.V). The selection, screening, and data extraction phases were independently performed to prevent bias. Any differences between researchers have been resolved by consulting an expert in this field (F.S). The data extracted from the included studies are first author, year of publication, country, and type of disease, design of the study, intervention, and type of intervention, number of centers/providers/patients, patient outcomes, provider outcomes, outcome impact, and statistical output.

### Quality assessment

We assessed the quality of studies by Jadad scale, the Oxford research methods scoring system for bias in clinical trials [21, 22]. We also determined the quality score by adding total scores for each sample. Meanwhile, we used zero and one for the exclusion/inclusion of items such as randomization, blindness, removal, dropouts, inclusion criteria, assessment of findings, and explanation of the statistical analysis.

### Data synthesis and statistical analysis

We extracted data from qualified articles using a structured data extraction method. The findings of the studies were presented in a descriptive-narrative form. In the meantime, we have conducted a meta-analysis with Comprehensive Meta-Analysis (CMA) statistical tools [23]. For all the analyzed data, the assessments of both the CDSS and the control groups were summarized as the mean standard deviation for each study and the comparison of pooled estimates between the intervention group and the control group. An effect size of std diff in the means of change in outcomes between groups was presented as standard error and 95% CI. The size of the effect with a lower limit greater than 0 implies that the intervention group has a positive effect on the outcome. The CDSS group does not affect the outcome compared to the control group when the lower limit is less than 0. Also, when std diff in means equals 0, it means that the change in outcomes was similar between the CDSS and the control groups. Meta-analysis using a random-effects model was performed to predict physician practice performance and patient outcomes. We used Q statistics and I2 to calculate heterogeneity (I2 greater than 50% is considered heterogeneous). Sensitivity analysis was also conducted to define and reduce the sources of heterogeneity. In the next step, the funnel plot was used to assess publication bias. A funnel plot is a valuable method for assessing potential visual publication bias [24].

## Results

On the basis of the inclusion criteria, we selected 45 qualified articles (Fig. 1). The assessment of the studies provided us with valuable information on the research goals, the types of electronic prescription systems, the types of diseases, and patients. Table 1 indicates that the findings of the quality evaluation of the studies were appropriate.

**Table 1:** Quality assessment for trials

References	Was research described as randomized?	Was approach of randomization appropriate?	Was research described as blinding?	Was approach of blinding appropriate?	Was there a presentation of withdrawal and dropouts?	Was there a presentation of the inclusion/exclusion criteria?	Was approach used to assess outcome?	Was the approach of statistical analysis described?	Total
Beeler et al. [25]	1	0	0	0	1	0	1	1	4
Eckman et al. [26]	1	1	0	0	1	1	1	1	6
Du et al. [27]	1	1	1	1	1	1	1	1	8
Karlsson et al. [28]	1	1	1	1	1	1	1	1	8
Mazzaglia et al. [29]	1	1	1	1	1	0	1	1	7
Nielsen et al. [30]	1	1	1	1	1	1	1	1	8
Patel et al. [31]	1	1	0	0	1	1	1	1	6
Akhu-zaheya et al. [32]	1	1	0	0	0	1	1	1	5
Khonsari et al. [33]	1	1	0	0	0	0	1	1	4
Christensen et al. [34]	1	1	1	1	1	1	1	1	8
Luitjes et al. [35]	1	1	1	1	0	1	1	1	7
Buhse et al. [36]	1	1	0	0	1	0	1	1	5
Perestelo-pérez et al. [37]	1	1	0	0	1	1	1	1	6
Sáenz et al. [38]	1	1	1	1	1	1	1	1	8
Vervloet et al. [39]	1	1	1	1	0	0	1	1	6
Vervloet et al. [40]	1	1	1	1	0	1	1	1	7
Geurts et al. [41]	1	1	0	0	1	0	1	1	5
Gill et al. [42]	1	1	0	0	1	1	1	1	6
Petersen et al. [43]	1	1	1	1	1	1	1	1	8
Bourgeois et al. [44]	1	1	1	1	1	1	1	1	8
Juszczyk et al. [45]	1	1	1	1	1	0	1	1	7
Mcdermott et al. [46]	1	1	1	1	1	1	1	1	8
Mcginn et al. [47]	1	1	0	0	1	1	1	1	6
Mohammed et al. [48]	1	1	1	1	0	1	1	1	7
Ackerman et al. [49]	1	1	0	0	1	0	1	1	5
Pop-eleches et al. [50]	1	1	0	0	1	1	1	1	6
Avansino et al. [51]	1	1	1	1	1	0	1	1	7
Awdishu et al. [52]	1	1	1	1	1	1	1	1	8
Erler et al. [53]	1	1	1	1	1	1	1	1	8
Cox et al. [54]	1	1	0	0	1	0	1	1	5
Muth et al. [55]	1	1	1	1	1	1	1	1	8
Strom et al. [56]	1	1	0	0	1	1	1	1	6
Strom et al. [57]	1	1	1	1	1	1	1	1	8
Elliott et al. [58]	1	1	1	1	0	0	1	1	6
Bruxvoort et al. [59]	1	1	1	1	0	1	1	1	7
Beeler et al. [60]	1	1	1	1	1	1	1	1	8
Duke et al. [61]	1	1	1	1	1	1	1	1	8
Eschmann et al. [62]	1	1	1	1	1	0	1	1	7
Curtain et al. [5]	1	1	0	0	1	1	1	1	6
Turchin et al. [6]	1	1	0	0	1	1	1	1	6
Griffey et al. [63]	1	1	0	0	1	1	1	1	6
Myers et al. [64]	1	1	1	1	1	1	1	1	8
Van Stiphout et al. [65]	1	1	1	1	1	1	1	1	8
Willis et al. [66]	1	1	1	1	0	1	1	1	7
Tamblyn et al. [67]	1	1	1	1	0	1	1	1	7
Total point earned									303
Quality Score									82.34

1 stands for the answer “yes”, and 0 stands for the answer “no”

The findings also demonstrated the effectiveness of CDSS in many diseases such as cardiovascular disease, high blood pressure, and diabetes, or cases such as simultaneous prescription of drugs. Findings from the analyzed studies are presented in Table 2 in which * stands for *p* values indicating a statistically significant difference.

**Table 2:** Data extracted for CDSS trials

References	Disease type	No. of hospitals/physicians/patients	Type of computer system	Outcome	*p* value
Beeler et al. [25]	Cardiovascular	–/–/15,736	Computerized system equipped with reminder to prevent intravenous thromboembolism	Increasing the ratio of prescribing prophylaxis 6–24 h after admission/transfer	*p value < 0/0001*
					*0/03
Eckman et al. [26]	Cardiovascular	15/–/1493	CDSS providing treatment recommendation	Reducing disagreement among physicians	*0/02
Du et al. [27]	Cardiovascular	58/–/patients	CDSS in mobile devices	Increasing secondary preventive prescriptions after 15 months in the intervention group	From 73/7 to 86/8 percent
Karlsson et al. [28]	Cardiovascular	43/–/14,134	CDSS equipped with alerts for patients with atrial fibrillation	Increasing the prescription of anticoagulation after 12 months	*0/01
Mazzaglia et al. [29]	Cardiovascular	–/197/–	Alert-based CDSS for patients using cardiovascular drugs	Increasing prescription of anti-blocking drugs	*p value < 0/001
Nielsen et al. [30]	Cardiovascular	–/–/191	CDSS to regulate the rate of warfarin use	Increasing the time outcome in the scope of treatment	0/67 Percent
Patel et al. [31]	Cardiovascular	23/178/–	Framework for the UK Medical Research Council (MRC)	Increasing the number of anti-inflammatory/lipid-lowering drugs	*p value < 0/001
Akhu-zaheya et al. [32]	Cardiovascular	–/–/160	Short message reminder system in adherence to a healthy nutritional diet, drugs, cessation of smoking	Increasing prescriptions in the short message group	*0/001
Khonsari et al. [33]	Cardiovascular	–/–/62	Web-based software equipped with text reminders for patients with chronic coronary syndrome	Increasing adherence to drug usage	*p value < 0/01
Christensen et al. [34]	Hypertension	–/–/398	Reminder in patient admission and blood pressure control	Reducing blood pressure after 12 months	0/06
Luitjes et al. [35]	Hypertension	16/–/532 at pre implementation phase,–/–/1762 at post implementation phase	Innovative strategy including decision support system, audit and feedback	For the control group, reducing the secondary outcome of infant morbidity after implementation	*p value < 0/0001
Buhse et al. [36]	Diabetes	22/–/363	ISDM-P program composed of CDSS and sessions	Reduction in faulty knowledge causing risk	*p value < 0/001
Perestelo-pérez et al. [37]	Diabetes	14/29/168	The CDSS selects statin with an estimate of cardiovascular disease risk	Increasing satisfaction of decision making	*0/009
Sáenz et al. [38]	Diabetes	66/–/697	The CDSS including patient data, glucose profile and recommendation for physician	Increasing long-term blood sugar using between group differences	*0/01
Vervloet et al. [39]	Diabetes	–/–/161	Real-time monitoring system for drug use by applying short message for diabetic patients	Increasing adherence in the group receiving short messages	*p value < 0/001
Vervloet et al. [40]	Diabetes	–/–/104	Real-time medication monitoring system equipped with short message reminder for patients with type two diabetes	Increasing the drug dosage in one hour during a six month period	*0/003
Geurts et al. [41]	Digestive diseases	–/–/222	Recommendation decision support system	Increasing the standard use of oral rehydration solution	*p value < 0/05
Gill et al. [42]	Digestive diseases	27/119/5234	CDSS equipped with alert functionality and integrated with electronic health record and clinical guidelines	Increasing the receiving care on the basis of instructions for patients with low-dose aspirin use (25%)	1/30
Petersen et al. [43]	Digestive diseases	General physicians	CDSS equipped with risk notification service	Increasing the drug prescription in patients with risk above 5 percent	*0/01
Bourgeois et al. [44]	Pulmonary diseases	–/112/–	Chronic obstructive pulmonary disease pattern in electronic health records	Reduced antibiotic prescriptions in visits by using templates	*0/02
Juszczyk et al. [45]	Pulmonary diseases	–/79/–	Electronic health records combined with databases of Electronic medical records such as links to clinical practice research data	Reducing unnecessary prescription of antibiotics	*0/04
Mcdermott et al. [46]	Pulmonary diseases	–/103/–	DSS and electronic learning	Increasing physicians self-efficacy	*0/02
Mcginn et al. [47]	Pulmonary diseases	–/–/984	A real time and unified CDSS during care combined with integrated clinical prediction rules	Reduced antibiotic prescription	*0/008
Mohammed et al. [48]	Pulmonary diseases	–/–/2207	Short message as a two-way reminder	Inability to be effective in treatment success rate	0/76
Ackerman et al. [49]	Pulmonary diseases	–/29/33	CDSS in Electronic Health Records	Reducing excess prescription of antibiotics	*0/003
Pop-eleches et al. [50]	Aids	–/–/428	Short-message reminder systems (daily and weekly) in the antivirus treatment process	Reducing the number of treatment interruptions in both groups receiving weekly messages	*0/02
Avansino et al. [51]	Appendicitis	–/7/–	Systematically developed order set for using the decision support system	Increasing the follow-up clinical guidelines for systematic prescriptions compared to case prescriptions	*0/003
Awdishu et al. [52]	Kidney diseases	–/514/1278	DSS WarninDSS Warnin	An increase in not taking medication or changing dose of inadequate drugs	*p value < 0/0001
Erler et al. [53]	Kidney diseases	–/44/404	Software including a database in coronary resection	Reduction in the amount of medication received in the intervention group in excess of the prescribed dose	*0/04
Cox et al. [54]	Taking multiple medications	–/–/216	The CDSS with medication order entry in order to determine the initial drug dosage	An increase in the number of prescriptions for initial drug use	*p value < 0/0001
				An increase in the conformity of prescribed medication percentage with the suggested medication	*p value < 0/00,001
Muth et al. [55]	Taking multiple medications	–/71/465	Reminder-based CDSS	Ineffectiveness of drug prescriptions after 6 and 9 months	0/31, 0/18
Strom et al. [56]	Taking multiple medications	–/1981/–	Computerized drug prescribing systems equipped with hard-alerted CDSSs	Increasing the percentage of appropriate alerts that have been responded to by physicians in the intervention group compared to the control group	57/2 versus 13/5
Strom et al. [57]	Taking multiple medications	–/1963/–	Computerized medication order entry system equipped with various alerts	Reduction in the appropriate response of physicians to alerts during 17 months	*0/007
Elliott et al. [58]	Taking multiple medications	–/–/110	Prescribing CDSS for creating drug treatment recommendations such as drug-drug and drug-gene interaction	Reducing the average number of days re-hospitalized 60 days after discharge	*0/007
				Reducing the combination of re-hospitalizations, emergency ward visits and morbidity 60 days after discharge	*0/005
Bruxvoort et al. [59]	Malaria	82/–/–	Text message reminders for Malaria treatment	Physicians’ knowledge in using Lumefantrine orthometer	*p value < 0/0001
Beeler et al. [60]	Increasing blood potassium	29/–/4861	Three types of CDSSs including reminder, high potassium and calcium alerts	An increase in the average monitoring time of potassium level	*p value < 0/001
Duke et al. [61]	Increasing blood potassium	–/1029/–	Drug-drug interaction alerts for patients in danger of high potassium level	A decrease in the conformity rate in normal risk patients for increased potassium	*p value < 0/01
Eschmann et al. [62]	Increasing blood potassium	15/–/37,000	Electronic health records equipped with alerts and reminders systems	A decrease in the reaction time of reminders for physicians monitoring alerts of potassium level	*0/04
Curtain et al. [5]	Medication prescription for the patient	185/–/–	CDSS for drug distribution in treatment with proton pump	Reduction in the approved percentage of inhibitor intervention proton pump which is registered by the pharmacologist	*p value < 0/001
Turchin et al. [6]	Medication prescription for the patient	–/3703/–	Hard alert systems to facilitate medication services	Increasing overall efficiency of system functionalities prior to admission	*p value < 0/0001
Griffey et al. [63]	Medication prescription for the patient	–/–/1407	CDSS for recommending drug dosage	Increasing the number of prescriptions by recommending the determined system dose	*p value < 0/0001
Myers et al. [64]	Medication prescription for the patient	–/59/–	Computerized alerts for manual or automatic correction of medical abbreviation	Reducing the significant number of inappropriate abbreviations	*0/02
Van Stiphout et al. [65]	Medication prescription for the patient	2/115/1094	CDSS integrated with training session	More efficient medical summary	*0/03
Willis et al. [66]	Medication prescription for the patient	–/–/2219	CDSS alerts for the primary care clinic	A lack of difference in the rate of patient adherence to treatment, drug treatment significance, economic and clinical outcomes in three groups	*0/01
Tamblyn et al. [67]	Mental disorders	–/81/5628	DSS equipped with three types of alerts	Reduction in dose of drugs after one year for antipsychotics	*0/02

**Fig. 2:**
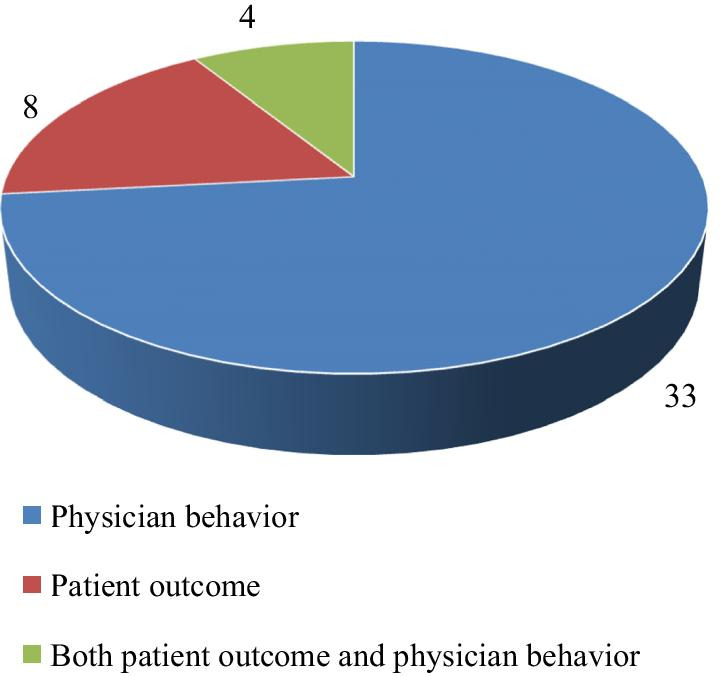
The number of studies based on several evaluating outcomes. The number of studies that assessed different kinds of outcomes based on patient outcomes, physician performance, or both outcomes is identified

**Fig. 3:**
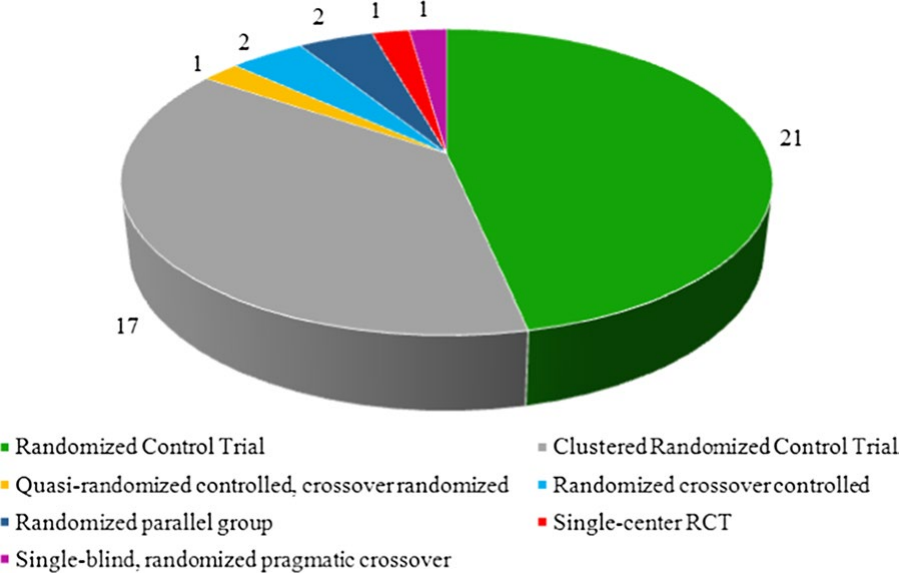
The number of studies based on the type of included studies. The number of studies focused on different types of randomized controlled trials has been established

The number of studies based on multiple evaluation results and types of studies is also shown in Figs. 2 and 3, respectively. Table 2 shows the variety of outcomes for several medication scopes (for example, the outcome "Increasing the ratio of prescribing prophylaxis" is specific for cardiovascular domain, or the outcome "Reducing blood pressure" is related to hypertension disorders). Meanwhile, Table 2 shows various kinds of CDSSs for prescribing classified according to alerts, reminders, recommendations, instruction, and a combination of these types. Table 2 also briefly presents the outcome of the thirteen medication scopes involved.

### The effect of CDSS on cardiovascular diseases

For patients admitted to the hospital, the level of venous thromboembolism prophylaxis, and the proportion of prescribed prophylaxis increased during 6–24 h after admission [25]. In another study, the discrepancies among physicians over the thromboprophylaxis treatment decreased with the aid of CDSS by offering treatment recommendations (*p* = 0.02) [26]. In other studies, alert-based CDSSs have positive effects on physician performance and treatment improvement in anti-inflammatory and lipid-lowering drugs [28, 29, 31]. By following medical recommendations in another study, physicians in the intervention group were able to improve the prescribing level of secondary preventive medication through using a regular CDSS [30]. Also, in other trials, the short messages of the program had a positive effect on patient adherence to medication and diet (*p* < 0.01) [32, 33].

### The effect of CDSS on hypertension

In one study, the electronic monitoring and recall program had no effect on blood pressure reduction and the admission of patients [34]. However, in another study, the patient outcome improved following the implementation of the CDSS [35].

### The effect of CDSS on diabetes

In some studies, the Real-Time Medication Monitoring (RTMM) system, equipped with a short message reminder, improved the precision of patients’ compliance and missed dose [36, 37, 39, 40]. In another study, HbA1c and group differences were greater in the intervention group using recommendation CDSS than that of the control group [38]. The use of statins (*p* = 0.03) and the problem areas in diabetes (PAID) (*p* = 0.01) improved in another study for the intervention group that used CDSS [37].

### The effect of CDSS on digestive diseases

In all studies, the CDSS had an effect on prescribing non-steroidal anti-inflammatory drugs, proton pump inhibitors, and increasing the standard use of oral rehydration solution without any difference in other results [41–43]. Also, alert-based CDSS improved the quality of patient care in another study [42].

### The effect of CDSS on pulmonary diseases

In some trials, the use of CDSS which was integrated with electronic health record or prediction rules resulted in a decrease in the prescribing of antibiotics and macrolides; therefore, it helped minimize the inappropriate use of antibiotics (*p* = 0.0005), reduce the resistance to antibiotics (*p* = 0.04), and enhance primary care [44–47, 49]. The patients adhered to the reminder message in another study; however, the messages did not affect the success rate of therapy [48].

### The effect of CDSS on AIDS

Results of this study showed that the reminder system for short text messages had a positive effect on the treatment process. Also, the number of messages did not have a significant effect on patients’ compliance rates (*p* = 0.12) [50].

### The effect of CDSS on appendicitis

This study showed that the system's systematically developed order set, which used clinical guidelines, improved system usability (*p* = 0.05), and reduced system-related problems (*p* = 0.05). This is the result of Computerized Provider Order Entry (CPOE) which improved efficiency, quality, and safety [51].

### The effect of CDSS on kidney diseases

One study showed the positive effect of the multipurpose intervention on creatinine value estimation and dose adjustment to reduce the insufficient dosage of primary care drugs [53]. In another study, the appropriate prescription rate for kidney problems was low, as opposed to the results of the former study. Also, the effectiveness of the CDSS with physician guidelines has been improved [52].

### The effect of CDSS on taking multiple medications

In one study, CDSSs resulted in delayed drug treatment for four patients needing urgent treatment. This suggests that the adverse effects of these systems need to be evaluated and monitored [56]. In another study, the CDSS improved the primary dose of medication, time intervals for drug use, and drug concentration which is to be injected intravenously compared to standard doses [54]. Also in another study, the average number of readmission days for each patient and the combination of re-hospitalization and emergency ward visits within 30 days after hospital discharge did not vary between the intervention group using recommendation CDSS and control groups [58]. In some trials, there was no discrepancy between the outcomes of the dosage rate and the Modified Medication Appropriateness Index (MMAI).

In the meanwhile, no discrepancy was seen among improper medication prescribing (*p* = 0.48), the Medication Regimen Complexity Index, and the mean pain outcome difference after 6 months (*p* = 0.13) and 9 months (*p* = 0.78) between the intervention group using alert or reminder CDSS and the control group [55, 57].

### The effect of CDSS on Malaria

The use of text-messaging in one study did not affect the patients’ behavior in completing the course of medication for the full duration of treatment. However, when the side effects were low (*p* = 0.02), it had some effects on the continuous use of the medication. In addition, text messages had an effect on physicians’ knowledge about the use of medications with fatty foods. (*p* < 0.0001) [59].

### The effect of CDSS on increasing the level of blood potassium

In one study, there is no statistical difference between the control and intervention groups in terms of following alerts and patients’ compliance rate. However, the physicians’ compliance rate improved at the medium potassium level from 3 to 3.9 (mili-equivalents/liter) (*p* < 0.01) [61]. Due to the rapid response of physicians to program alerts for high potassium levels in the intervention group, the positive effect of the system on physician performance was evident in another study (*p* = 0.01) [62]. However, in another study in this section, the time-lapse in hyperkalemia monitoring (*p* = 0.20) and the incidence rate of hyperkalemia (*p* = 0.22) did not vary significantly even with the use of three different kinds of reminder and alert-based CDSSs [60].

### The effect of CDSS on medication prescription for patients

Based on the results of some studies, the regular or alert based CDSSs resulted in better drug prescriptions for the proton pump inhibitor and a reduction in abbreviation prescriptions [5, 64]. Also, in other studies, the overall utilization of system functionalities, system utilization between two-time laps (*p* < 0.0001), number of users (*p* < 0.0001), and physicians’ compliance with the medication recommendations provided by the CDSS improved medication prescriptions which eventually resulted in reduced side effects (*p* = 0.02) [6, 63]. There was no difference in prescribing among physicians in one study (*p* = 0.14); however, the percentage of skilled questions for the intervention group equipped with training CDSS (*p* = 0.01) improved [65]. In another study, alert-based CDSSs have been effective in identifying evidence-based pharmacotherapies (EBP). In the meantime, compliance with treatment by health care managers has had no effect on patient outcome [66].

### The effect of CDSS on mental disorders

CDSS alerts resulted in reduced risk of injury and reduced dose of antipsychotics and anticoagulants (*p* = 0.03) over a one-year period. Therefore, CDSS reduced the risk of injury (*p* = 0.02) [67].

### Statistical and sensitivity analysis

**Fig. 4:**
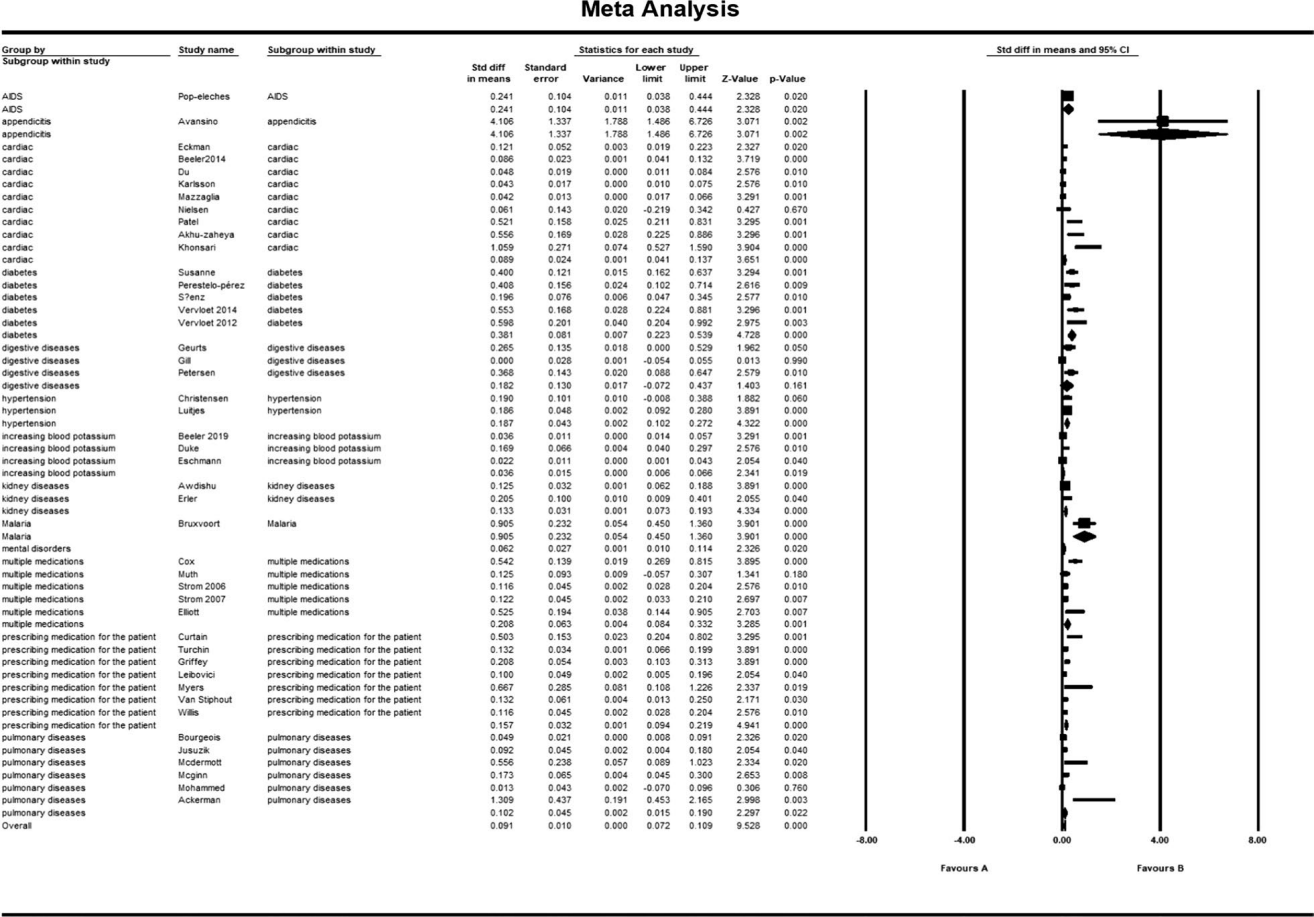
Forest plot of the overall effect of CDSS for prescribing on physician practice performance and patient outcome based on medication subgroup analysis. Meta-analysis is conducted using Comprehensive Meta-Analysis (CMA) statistical tools. The pooled std diff in means of *p* values showed a significant difference between the CDSS and the control group (std diff in means = 0.091, 95% CI 0.072–0.109, standard error = 0.010). Confidence Interval (CI) represents for the linear area between lower and upper limits

**Fig. 5:**
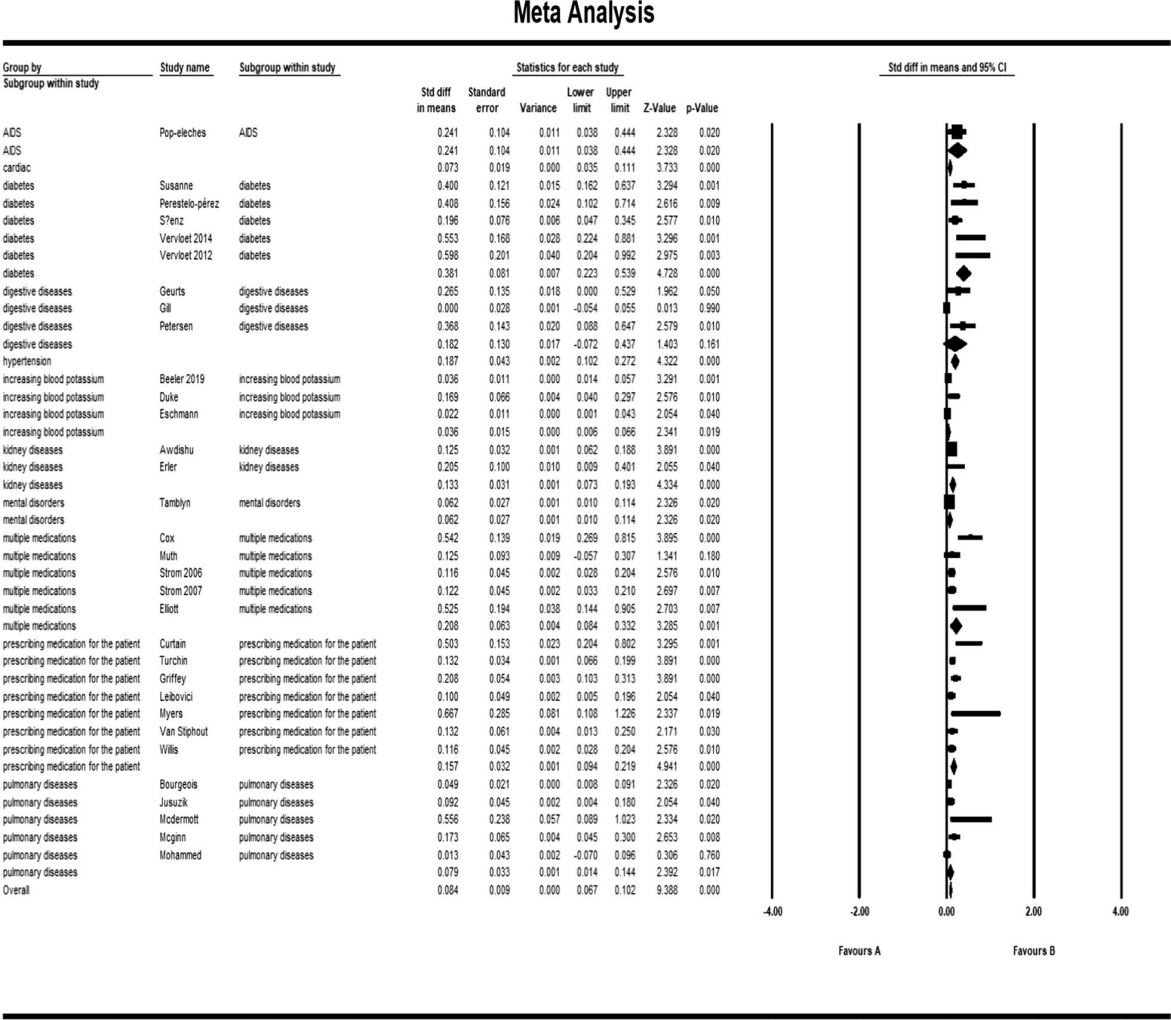
Forest plot of the overall effect of CDSS for prescribing on physician practice performance and patient outcome based on medication subgroup analysis after sensitivity analysis. After sensitivity analysis, heterogeneity improved considerably, excluding khonsari et al. [33]; Ackerman et al. [49]; Avansino et al. [51], and Bruxvoort et al. [59]. The pooled std diff in means of *p* values was used for evaluating the overall and subgroup effects of CDSS which were significantly different (std diff in means = 0.084, 95% CI 0.067–0.102) as a whole. Meta-analysis results for each subgroup of medication scope showed a significant difference between CDSS and control groups for medication scopes namely as hypertension (CI 0.102–0.272); increasing blood potassium (CI 0.006–0.066); multiple medications (CI 0.084–0.332); AIDs (CI 0.038–0.444); kidney disorders (CI 0.073–0.193); diabetes (CI 0.223–0.539); cardiac (CI 0.035–0.111); mental disease (CI 0.010–0.114); medication prescription (CI 0.094–0.219); and pulmonary disease (CI 0.014–0.144)

The pooled std diff in means of *p* values showed a significant difference between the CDSS and the control group (std diff in means = 0.091, 95% CI 0.072–0.109, standard error = 0.010). 95% CI for the effectiveness was drawn for each study in the horizontal line format (Q = 209.2, df = 45, *p* = 0.0002, I2 = 78.492, Tau2: 0.004) (Fig. 4). Due to the high heterogeneity of results, a sensitivity analysis was performed. In doing so, we excluded the following studies: khonsari et al. [33]; Ackerman et al. [49]; Avansino et al. [51], and Bruxvoort et al. [59]. Because of the limited number of patients in these trials, we decided to exclude them from our meta-analysis. In Tables 2 and 3, the characteristics of these studies are presented in narrative results. The findings indicate that heterogeneity improved considerably after sensitivity analysis (Fig. 5). (Q = 164.8, df = 41, *p* = 0.0001, I2 = 75.136, Tau2: 0.003). The overall effect of CDSS for prescribing medications on patient outcomes and physician practice performance based on the random-effects model was statistically significant (std diff in means = 0.84, 95% CI 0.067–0.102).

**Table 3:** Outcome classification for trials

References	Primary outcome	Outcome summarization	Outcome impact	Outcome category
Beeler et al. [25]	Increasing the ratio of prescribing prophylaxis 6–24 h after admission/transfer	Increasing prescribing	+	Physician practice performance improved
Eckman et al. [26]	Reducing disagreement among physicians	Reducing disagreement among physicians	+	
Du et al. [27]	Increasing secondary preventive prescriptions after 15 months in the intervention group	Increasing prescribing	+	
Karlsson et al. [28]	Increasing the prescription of anticoagulation after 12 months	Increasing prescribing	+	
Mazzaglia et al. [29]	Increasing prescription of anti-blocking drugs	Increasing prescribing	+	
Patel et al. [31]	Increasing the number of anti-inflammatory/lipid-lowering drugs	Increasing prescribing	+	
Perestelo-pérez et al. [37]	Increasing satisfaction of decision making	Increasing satisfaction of decision making	+	
Sáenz et al. [38]	Increasing long-term blood sugar using between group differences	Increasing prescribing	+	
Geurts et al. [41]	Increase in standard use of oral rehydration solution	Increasing prescribing	+	
Petersen et al. [43]	Increase in drug prescription in patients with risk above 5 percent	Increasing prescribing	+	
Bourgeois et al. [44]	Reduced antibiotic prescriptions in visits by using templates	Reducing prescribing	+	
Juszczyk et al. [45]	Reducing unnecessary prescription of antibiotics	Reducing prescribing	+	
Mcdermott et al. [46]	Increasing physicians self-efficacy	Increasing physicians efficacy	+	
Mcginn et al. [47]	Reduced antibiotic prescription	Reducing prescribing	+	
Avansino et al. [51]	Increase in following clinical guidelines for systematic prescriptions compared to case prescriptions	Increase in following clinical guidelines	+	
Awdishu et al. [52]	Increase in not taking medication or changing dose of inadequate drugs	Reducing prescribing	+	
Erler et al. [53]	Reduction in the amount of medication received in the intervention group in excess of the prescribed dose	Reducing prescribing	+	
Cox et al. [54]	Increase in the number of prescriptions for initial drug use	Increasing prescribing	+	
Strom et al. [56]	Increasing the percentage of appropriate alerts that have been responded to by physicians in the intervention group compared to the control group	Increasing the percentage of appropriate alerts	+	
Beeler et al. [60]	Increase in the average monitoring time of potassium level	Increase in the average monitoring time of potassium level	+	
Eschmann et al. [62]	Decrease in the reaction time to reminders in physicians for monitoring alerts for potassium level	Decrease in the reaction time to reminders	+	
Curtain et al. [5]	Reduction in the approved percentage of inhibitor intervention proton pump which is registered by the pharmacologist	Reduction in the approved percentage of inhibitor intervention proton pump which is registered by the pharmacologist	+	
Turchin et al. [6]	Increasing overall efficiency of system functionalities prior to admission	Increasing overall efficiency of system functionalities	0	
Griffey et al. [63]	Increasing the number of prescriptions by recommending the determined system dose	Increasing prescribing	+	
Myers et al. [64]	Reducing the significant number of inappropriate abbreviations	Reducing prescribing	+	
Van Stiphout et al. [65]	More efficient medical summary	More efficient medical summary	+	
Akhu-zaheya et al. [32]	Increasing prescriptions in the short message group	Increasing prescribing	+	Patient outcome improved
Khonsari et al. [33]	Increasing adherence to drug usage	Increasing adherence	+	
Vervloet et al. [39]	Increasing adherence in the group receiving short messages	Increasing adherence	+	
ervloet et al. [40]	Increasing the drug dosage in one hour during a six month period	Increasing prescribing	+	
Elliott et al. [58]	Reducing the average number of days re-hospitalized 60 days after discharge	Reducing the average number of days re-hospitalized	+	
Bruxvoort et al. [59]	Knowledge of the physician in using Lumefantrine or thometer	Increased Knowledge of the physician	+	
Tamblyn et al. [67]	Reduction in dose of drugs after one year for antipsychotics	Reducing prescribing	+	
Luitjes et al. [35]	For the control group, reducing the secondary outcome of infant morbidity after implementation	Reducing morbidity	+	Physician practice performance and patient outcome improved
Ackerman et al. [49]	Reducing excess prescription of antibiotics	Reducing prescribing	+	
Pop-eleches et al. [50]	Reducing the number of treatment interruptions in both groups receiving weekly messages	Effective in process of care	+	
Christensen et al. [34]	Reducing blood pressure after 12 months	Reducing morbidity	0	Physician practice performance not improved
Nielsen et al. [30]	Increasing the time outcome in the scope of treatment	Increasing the time outcome	0	
Buhse et al. [36]	Reduction in faulty knowledge causing risk	Reducing risk	0	
Gill et al. [42]	Increase in receiving care on the basis of instructions for patients with low-dose aspirin use (25%)	Increase in receiving care	0	
Muth et al. [55]	Ineffectiveness of drug prescriptions after 6 and 9 months	Ineffectiveness in process of care	0	
Strom et al. [57]	Reduction in the appropriate response of physicians to alerts during 17 months	Reduction in the appropriate response of physicians to alerts	0	
Duke et al. [61]	Decrease in the conformity rate in normal risk patients for increased potassium	Decrease in the conformity rate in normal risk patients	0	
Willis et al. [66]	Lack of difference in the rate of patient adherence to treatment, drug treatment significance, economic and clinical outcomes in three groups	No difference in process of care outcomes	+	Patient outcome not improved
Mohammed et al. [48]	Inability to be effective in treatment success rate	Ineffectiveness in process of care	0

### Subgroup analysis for medication scope

**Fig. 6:**
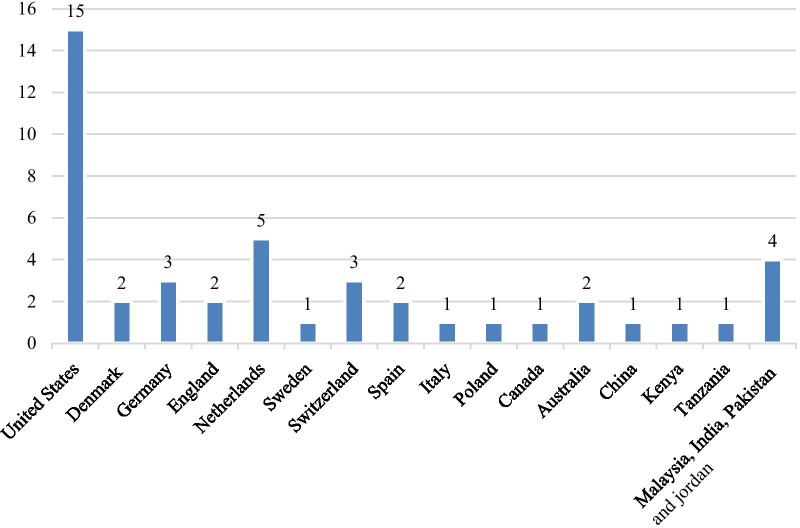
The number of studies associated with each country. The number of studies carried out in different countries is identified

**Fig.7:**
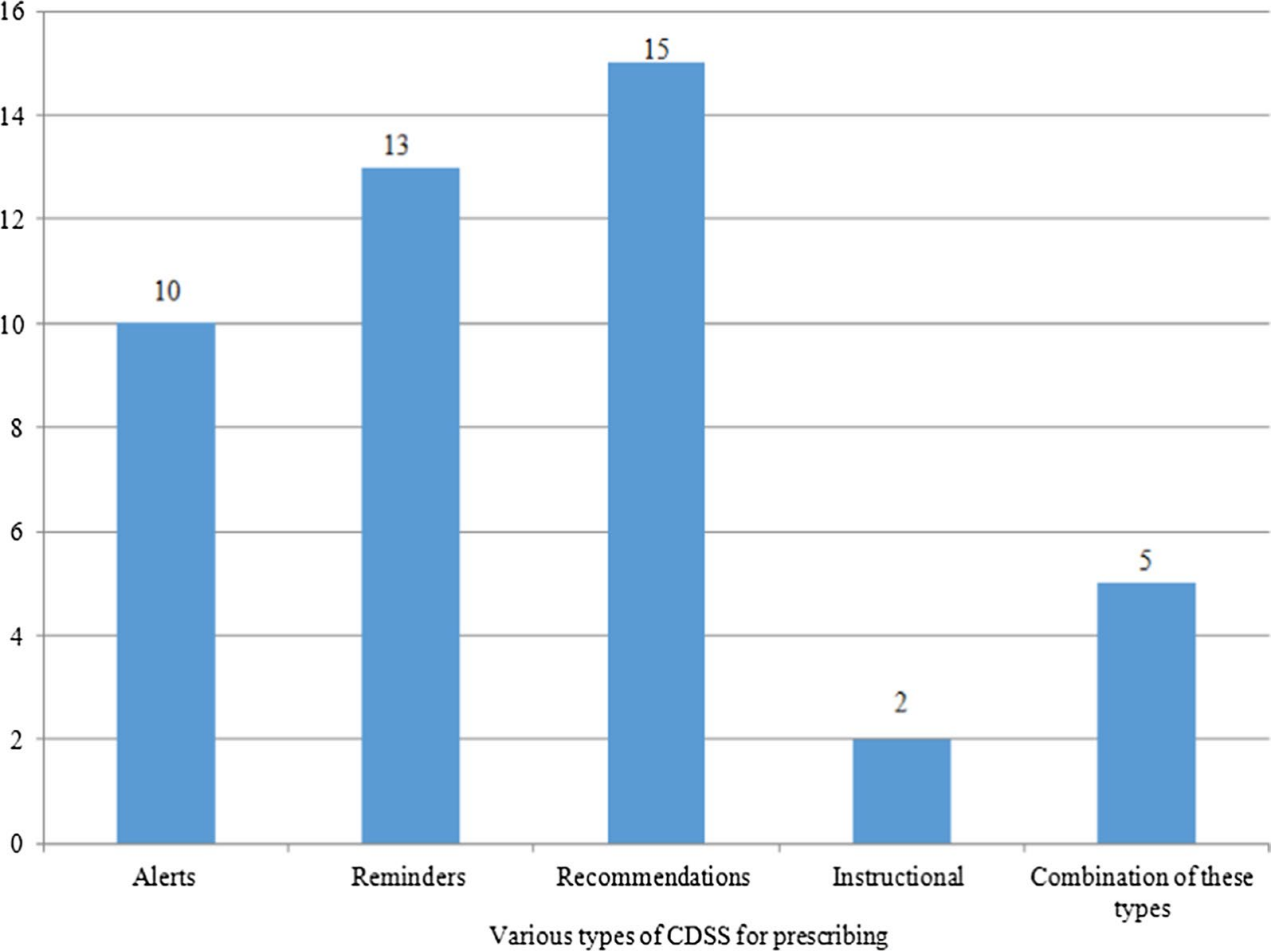
The number of studies associated with each CDSS type. The number of studies that were performed on various types of CDSS such as reminders and alarms is listed

Figure 5 shows the results of the meta-analysis for each subgroup of medication scope and the total analysis. Subgroup analysis is performed on different medication groups because there have been common outcomes in related similar medication scope studies. The subgroup analysis showed a significant difference between CDSS and control groups for medication scopes namely as hypertension: (std diff in means = 0.187, 95% CI 0.102–0.272); increasing blood potassium: (std diff in means = 0.036, 95% CI 0.006–0.066), multiple medications: (std diff in means = 0.208, 95% CI 0.084–0.332), AIDs: (std diff in means = 0.241, 95% CI 0.038–0.444), kidney disorders: (std diff in means = 0.133, 95% CI 0.073–0.193), diabetes: (std diff in means = 0.381, 95% CI 0.223–0.539), cardiac: (std diff in means = 0.073, 95% CI 0.035–0.111), mental diseases: (std diff in means = 0.062, 95% CI 0.010–0.114), medication prescription for patients: (std diff in means = 0.157, 95% CI 0.094–0.219), and pulmonary diseases: (std diff in means = 0.079, 95% CI 0.014–0.144). However, there was no significant difference between the intervention and control group for digestive diseases: (std diff in means = 0.182, 95% CI − 0.072 to 0.437). Figure 5 shows the forest plot for subgroup meta-analysis. However, we eliminated malaria and appendicitis diseases due to the decrease of heterogeneity among studies. We then described malaria and appendicitis diseases in narrative results. Also, Figs. 6 and 7 show the number of studies associated with each country and type of CDSS.

### Categorization of outcomes

Physician practice performance and patient outcomes are presented in Table 3 as primary outcomes are categorized based on the summary of the outcome concept and the impact of CDSS. Improvement or neutrality in outcomes is shown by plus or zero in Table 3. We categorized outcomes because similar outcomes may have different impacts on various diseases. For instance, the outcome “decrease prescribing” may have a positive effect on some diseases and no effect on other medication domains.

### Evaluation for publication bias

**Fig. 8:**
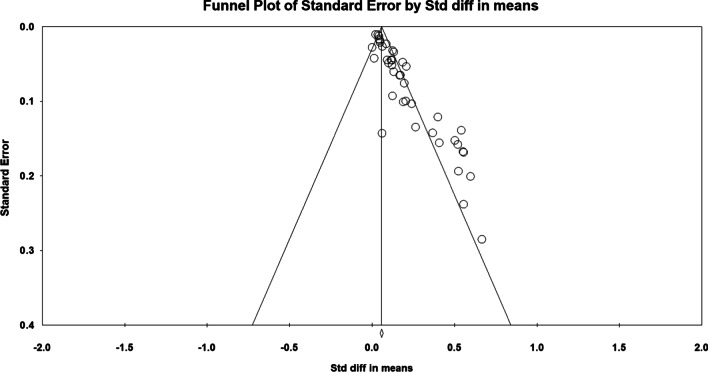
Funnel plot of standard error by std diff in means. There was no significant difference for publication bias for the included studies (*p* value = 0.000001). X-axis shows std diff in mean in the funnel diagram and the Y-axis reflects standard error. Dispersion of studies in the funnel plot showed that there was no bias in publication

We conducted a funnel plot and Egger’s regression to evaluate the publication bias regarding the effect of CDSS on patient outcomes and physician performance [68, 69]. There was no significant difference with respect to publication bias (std diff in means = 0.054, CI 95%: 2.116 to 2.941, *p* = 0.000001). Figure 8 depicts that the X-axis shows std diff in mean in the funnel diagram, and the Y-axis reflects standard error.

## Discussion

The aim of this systematic review is to establish the effect of CDSS on patient outcomes and physician performance. The effect of CDSS was measured using different methods in the included studies. In most cases, the effect of these programs on physician performance and patient outcomes were positive. In others, however, no significant effect has been found.

The results show that the use of CDSSs in cardiovascular patients has positive effects on physician performance by increasing the prescription of anticoagulants, anti-inflammatory drugs, anti-thrombotic drugs, lipid-lowering drugs, blood pressure drugs, cardiovascular drugs recommended for the reduction of cardiovascular diseases in patients with diabetes, and observing clinical guidelines [25, 27–29]. The results of the current study are consistent with the results of Duke et al. and Brokel et al. in reducing inadequate prescriptions and enhancing the process of observing clinical guidelines [61, 70]. Also, the system's user-friendliness environment and low running cost have resulted in its efficiency in the care delivery process [25, 27–29].

However, the results of our study have shown that using CDSSs for cardiac patients did not affect the physician performance in a number of outcomes such as physician conduct in prescribing drugs, the Warfarin treatment system, reducing frustration with antithrombotic diagnostic guidelines, and job satisfaction [26, 29–31]. The results of this study are also consistent with Byrnes and Lazaro studies in that clinical factors and treatment issues were the reasons for physicians' disagreement with system recommendations [71, 72]. The key explanation why there was no improvement in medical guidance is the complexity of clinical problems that could increase the risk of injury to the patient and delay the decision-making process [26, 29–31].

Also, the results of this study indicate that the use of CDSSs in cardiovascular patients has a positive effect on a number of outcomes such as adherence to drug use by patients and following a nutrition-based diet in the Mediterranean [32, 33]. Similarly, according to clinical guidelines and reminders, Schedlbauer et al.’s study reported the positive effect of CDSS on cardiovascular patient outcomes [73]. The reason for poor adherence to the Mediterranean diet was the delivery of a short message outlining the advantages of the Mediterranean diet which resulted in an improved conformity level [32, 33].

The study also showed that the use of CDSS in cardiovascular patients did not affect patient outcomes such as readmission rate, mortality, and smoking cessation [32, 33]. Similarly, the findings of Simpson et al.'s study indicate that accurate compliance with the Short Message System (SMS) reduces mortality risk and improves health outcomes [74]. One of the reasons for the negligible reduction in mortality is the short duration of the study, small sample size, and inability to identify causes of mortality [32, 33]. Also, study results show that the use of CDSS in patients with hypertension in adherence to clinical guidelines and laboratory tests has a positive effect on physician performance [35]. Zwart et al.'s study, which is consistent with the results of our study, assessed the impact of CDSS on adherence to clinical guidelines. The study reported effective results about the treatment of pregnant women with hypertensive disorders [75]. In addition, physicians’ awareness of special care during pregnancy for hypertension resulted in improved patient care and adherence to CDSS [35].

Based on the results of this research, the use of CDSS in diabetic patients has a positive effect on physician performance in a variety of outcomes such as adjusting the form of insulin and improving the quality of decision-making about statin prescription [36–38]. The findings of Den Ouden et al.’s and Mann et al.’s studies are also consistent with the results of our review which suggest physicians' strong adherence to CDSS, enhanced statin prescribing, and improved quality of medical care [76, 77]. In fact, the CDSS dynamically recommends the insulin dose based on the rounds of previous days, the type of insulin injected, and the glucose level of the patient on the day before [36–38].

The results of this study indicate that the use of CDSS in diabetic patients has a positive effect on a variety of patient outcomes such as adherence to the nutritional diet of patients with type 2 diabetes and taking the missed dose of medication [39, 40]. Meanwhile, the results of this study are consistent with Vervloet et al.’s and Krishna et al.'s systematic review on the positive effect of CDSS with alerts on patients with diabetes [78, 79]. The main reason for the effect of CDSS on improving patient adherence seems to be due to the fact that it raises patients’ awareness of taking medication [39, 40].

Also, the results of this study show that the use of CDSS in digestive disorders has a positive effect on the physician performance in a variety of outcomes such as the standard use of oral rehydration solution, the prescription of non-steroidal anti-inflammatory drugs and proton pump inhibitors in normal and high-risk patients, and the provision of care services in line with the guidelines for primary care [41–43]. The results of this study are also consistent with the findings of Nicastro’s study which stated that the system had positive effects on physician performance such as adherence to clinical guidelines and prescription of drugs [80]. The reason for the positive effect of CDSS on the prescription of non-steroidal anti-inflammatory drugs and proton pump inhibitors in high-risk patients and the use of oral rehydration solution was the systems’ recommendations about the above-mentioned drugs [41–43].

The results of this study also showed that the use of CDSS in respiratory patients had a positive effect on physician performance and reduced antibiotic prescription [44–47, 49]. The results of this study are therefore consistent with the findings of Mcdermott et al.’s and Butler et al.’s results on the positive effect of CDSS on the self-efficacy of physicians in managing chronic respiratory patients and reducing the prescription of antibiotics [81, 82]. We think that the reason for the system's positive effect on the self-efficacy of physicians was their tendency to cooperate on decision-making and not to receive mandatory CDSS recommendations [44–47, 49].

With respect to respiratory patients, the results of this study show that the use of CDSS has a positive effect on some patient outcomes such as reduced antibiotic resistance and a reduction in antibiotic prescription [48, 49]. Similarly, the results of Hebert et al.’s and Steinman et al.’s studies show reduced resistance to antibiotics [83, 84]. We conclude that the patient-physician partnership with the CDSS guideline, which played a significant role in the prescribing of medicines, was the explanation for the positive effect of CDSS on the reduction of irrational antibiotic prescription and resistance [48, 49].

With respect to appendicitis, the results of our review indicate that the use of CDSS has a positive effect on physician performance in certain outcomes such as performance, quality, and safety with the assistance of physicians’ computerized order entry [51]. The results of this review are in line with Holden's study which explores how physicians using the order entry system would receive more up-to-date information and boost the system's capabilities [85]. Although prescriptions are not strong in terms of content, errors are decreased as CPOE encourages physicians to consider cases [51].

Also, the results show that the use of CDSS in kidney patients has a positive effect on physicians' performance in some outcomes such as reduced dosage of inadequately prescribed drugs and the improved rate of adequate prescription [52, 53]. Such findings are consistent with Bates et al.’s and Chertow et al.’s studies which show the positive effect of CDSS alerts on modifying insufficient prescriptions and increasing the recommended level of inadequate dosage [86, 87]. The timeline of CDSS alerts was the main reason for the positive impact of CDSS on the prescription and recommended dosage of drugs [52, 53].

Based on the results of our review, the use of CDSS in patients with high blood potassium levels has a positive effect on physicians' performance in some outcomes such as the faster physicians’ response in the intervention group to system alerts and reminders [62]. is study are also consistent with Helmous et al.’s and Paterno et al.’s reports which show that physicians’ adherence to alerts improved by 19 percent [88, 89]. The key explanation for the positive effect of CDSS on physician performance was uninterrupted alerts and reminders [62].

Results of the study showed that the use of CDSS in prescribing drugs for patients has a positive effect on physician performance in certain outcomes such as drug prescription for proton pump inhibitors, CDSS productivity and usability, reduction of drug side effects, and improving the learning rate and physicians’ skills [5, 6, 63–65]. The results of this study are consistent with the results of Curtis and Shah et al.’s study indicating that relevant CDSS, while providing users with performance-related information, reduces patients' harms and errors, and increases physicians’ knowledge and skills [90, 91]. One of the main reasons for the proton pump's enhanced medication performance was the control of prescription drug dose by physicians as well as equipping pharmacies with CDSS with hard alerts which reduce costs and improve usability [5, 6, 63–65].

Results show that the use of CDSS in prescribing a number of drugs has a positive effect on physician performance in some outcomes such as the number of emergency ward visits, the number of re-hospitalizations, and determination and supervision of the number of drugs including the initial dose [54, 58]. The results are consistent with Vincent and Cordero's study which demonstrates that combining the computerized order entry process with an alert system saves time in prescribing and optimizing the dosage of drugs [92, 93]. The reason for CDSS' positive effect on the number of re-hospitalizations, emergency ward visits, and reduced morbidity rate was due to the fact that CDSS had a knowledge base in pharmacogenetics and was equipped with drug interaction alerts [54, 58].

Analysis of the results of the reviewed studies shows that the use of CDSS in prescribing a number of drugs has no effect on the physician performance in outcomes such as drug prescription rates with drug suitability index, average functional status outcome, and drug complexity index [55–57]. The results of our study are consistent with Olsson’s study which shows that CDSS for elderly people, who use multiple types of medicines, has no effect on important outcomes [94]. We conclude that the unexpected findings of our review may be due to the lack of information about patients with serious infections who require immediate care and the lack of an efficient checklist monitoring the patients’ drug problems [55–57].

The most critical CDSS system factors for outcome improvement are: alignment of guidelines with registered and EHR data to make decisions about each individual patient [29, 31, 33]; the short massages that include only necessary alerts such as drug interaction alerts sent in the right time for prescribing and user-friendly interface for saving physicians’ time [18, 20, 44, 45, 47, 48]; giving the choice to users by enabling them to close the alert window and move through next steps or provide uninterrupted alerts [37–41, 56]; the behaviors of physicians and patients which have positive effects on outcomes in CDSS-equipped environments through collaboration, following guidance, recommendations, alerts, and reminders that the system provides [37, 42]. Also, considering physician perception in defining the importance of alerts helps better understand the interruption status of alerts [37–41].

### Subgroup analysis for CDSS types

**Fig. 9:**
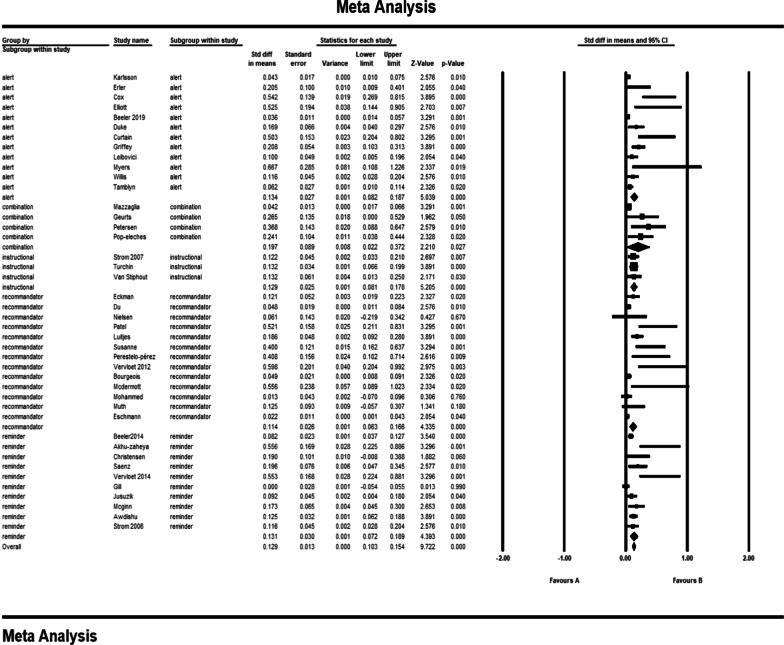
Forest plot of the effect of CDSS for prescribing on physician practice performance and patient outcome based on subgroup analysis for CDSS types. The subgroup analysis for various CDSS types showed a significant difference for alerts (CI 0.082–0.0187); combination types of CDSSs (CI 0.022–0.372); recommendation CDSSs (CI 0.063–0.166); reminders (CI 0.072–0.189); and instructional CDSSs (0.081 to 0.178). The results are assessed following the exclusion of khonsari et al. [33]; Ackerman et al. [49]; Avansino et al. [51] and Bruxvoort et al. [59] studies

The subgroup analysis for various CDSS types showed a significant relationship between CDSS and control group for alerts: (std diff in means = 0.134, 95% CI 0.082–0.0187); combination types of CDSSs: (std diff in means = 0.197, 95% CI 0.022–0.372); recommendation CDSSs: (std diff in means = 0.114, 95% CI 0.063–0.166); reminders: (std diff in means = 0.131, 95% CI 0.072–0.189); and instructional CDSSs: (std diff in means = 0.129, 95% CI 0.081–0.178). Figure 9 shows the forest plot for CDSS types.

### Outcome analysis

**Fig. 10:**
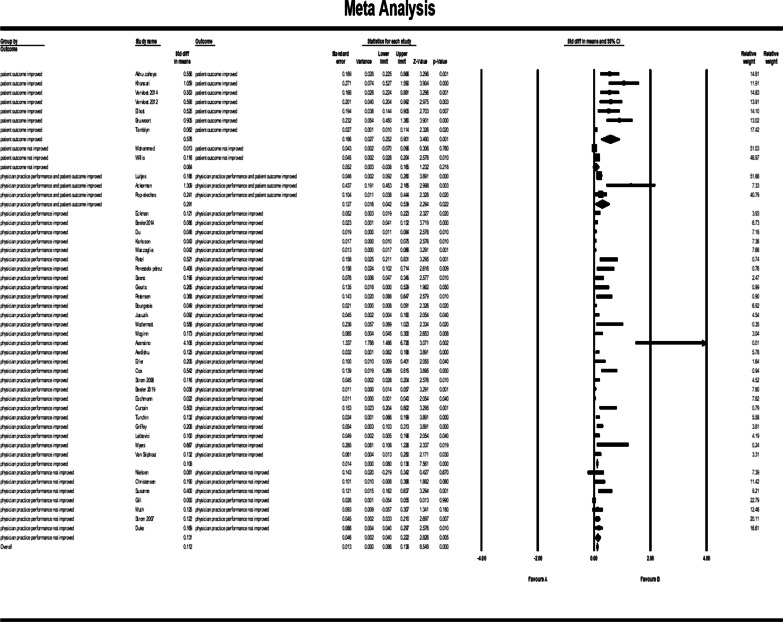
Forest plot of the overall effect of CDSS for prescribing on physician practice performance and patient outcome based on outcome categorization. The pooled std diff in the mean *p* values did not indicate a significant difference between the CDSS and the control group before the sensitivity analysis was performed (std diff in means = 0.0110, 95% CI 0.086–0.138, standard error = 0.013)

**Fig. 11:**
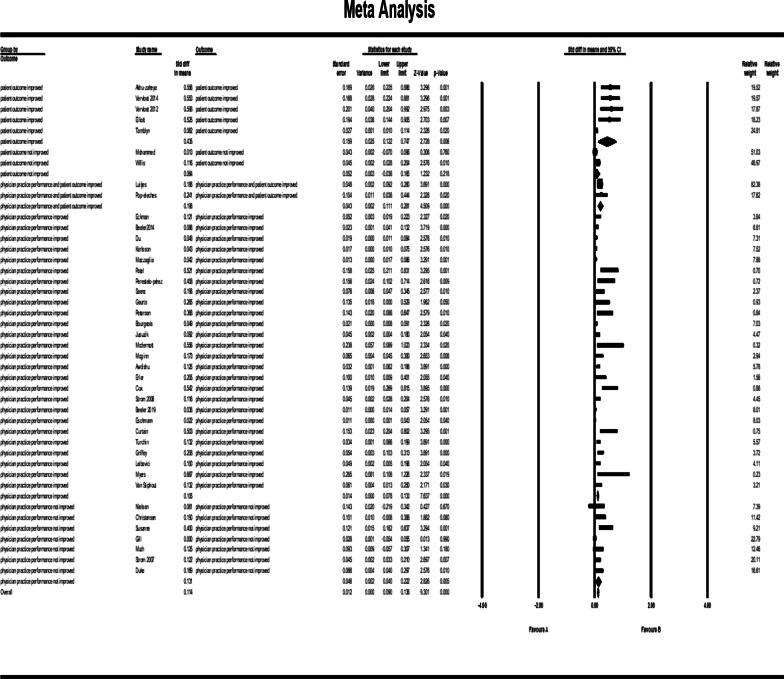
Forest plot of the overall effect of CDSS for prescribing on physician practice performance and patient outcome based on outcome categorization. The overall effects of prescribing CDSS on patient outcomes and physician practice performance after performing sensitivity analysis were significantly different: (std diff in means = 0.114, 95% CI 0.090–0.138). The outcome analysis showed a significant difference between CDSS and the control group for outcome categories such as patient outcomes improved (CI 0.122–0.747); physician practice performance improved (CI 0.78–0.133); physician practice performance and patient outcomes improved (CI 0.111–0.281); and physician practice performance didn’t improve (CI 0.040–0.222). There was not a significant difference in the category of ‘not improved’ for patient outcomes (CI − 0.038 to 0.165). The results are assessed following the exclusion of khonsari et al. [33]; Ackerman et al. [49]; Avansino et al. [51] and Bruxvoort et al. [59] studies

Figure 10 shows the results of meta-analysis for outcome categories and the total analysis. The pooled std diff in means of *p* values did not show a significant difference between CDSS and the control group (std diff in means = 0.0110, 95% CI 0.086–0.138, standard error = 0.013). 95% CI for the effectiveness was drawn for each study in the horizontal line format (Q = 209.2, df = 45, *p* = 0.0003, I2 = 78.492, Tau2: 0.004). The findings indicate that heterogeneity improved considerably after sensitivity analysis (Fig. 11). (Q = 164, df = 41, *p* = 0.0002, I2 = 75, Tau2: 0.003). After this change, the overall effects of clinical decision support system for prescribing on patient outcomes and physician practice performance based on the random effect model was significantly different: (std diff in means = 0.114, 95% CI 0.090–0.138).

The outcome analysis showed a significant difference between CDSS and control groups for the categorization of outcomes. Results showed that patient outcome improved: (std diff in means = 0.435, 95% CI 0.122–0.747); physician practice performance improved: (std diff in means = 0.105, 95% CI 0.78–0.133); physician practice performance and patient outcome improved: (std diff in means = 0.196, 95% CI 0.111–0.281); physician practice performance didn’t improve: (std diff in means = 0.131, 95% CI 0.040–0.222). The outcome analysis did not confirm a significant difference between CDSS and control groups for the category of patient outcome: (std diff in means = 0.064, 95% CI − 0.038 to 0.165).

The CDSS types that have enhanced the outcome for patients or physician practice are as follows: alerts, recommendations, instructional CDSSs, reminders, and a combination of all of them. Patient outcomes and practice performance outcomes have been improved with the use of the CDSSs for prescribing. In some trials, however, the CDSS was not specifically related to patient outcomes and showed only a marginal improvement in medical practice outcomes.

### Limitations and implications for research

Although we conducted a meta-analysis on the outcomes based on subgroup analysis, the heterogeneity among the included studies in our analysis prevented us from using sturdier mix methods. The effect that we expected of the system as a whole was statistically significant. Since we used the main outcome data for meta-analysis of the trials, there could be other outcomes by choosing certain secondary outcomes that are not statistically different from our findings. Further outcomes can be obtained by extending the spectrum of all kinds of CDSSs in addition to CDSS for prescribing.

## Conclusion

This systematic review study was conducted with the aim of identifying the effect of CDSS on patient outcomes and physician performance. The results show that the use of CDSS in some diseases has positive effects on patient outcomes and physician performance while it has no significant effect on others. In addition, the types of outcomes and the effects of CDSS on the diseases are different. In some cases, the use of this approach yields positive outcomes for patients and physicians; however, in some other cases, it shows no significant difference compared to conventional approaches. The positive effect of CDSS seems to be attributed to factors such as the user-friendliness of the system, the number of patients requiring treatment, the rate of observance of clinical guidelines, the conformity of clinical guidelines and data registry, the rate of patients’ accurate adherence to messages of the system, the usefulness of short messages, the existence of algorithms with dynamic functioning based on patient data, the existence of patient medical records, the relationship between electronic health records with CDSS and timely alerts of the system in the prescribing process. In addition, the positive effect of CDSS depends on a number of other factors such as providing an instruction section, not being confronted with mandatory recommendations, patient and physician cooperation with the aid of CDSS guidelines, not lagging between alerts where the alert is of low importance, the identification of important alerts, equipping pharmacies with CDSS and system applicability, and considering the opinions of physicians when assessing the value of alerts for drug interaction.

## Data Availability

All data generated or analyzed in the course of this study are included in this article.

## Abbreviations

CDSS: Clinical Decision Support System
MeSH: Medical Subject Headings
CPOE: Computerized Physician Order Entry
PRISMA: Preferred Reporting Items for Systematic Reviews and Meta-Analyses
MRC: UK Medical Research Council
RTMM: Real-Time Medication Monitoring system
PAID: Problem Areas in Diabetes
MMAI: Modified Medication Appropriateness Index
SMS: Short Message System
RCT: Randomized Controlled Trial
